# Documenting Social Media Engagement as Scholarship: A New Model for Assessing Academic Accomplishment for the Health Professions

**DOI:** 10.2196/25070

**Published:** 2020-12-02

**Authors:** Kimberly D Acquaviva, Josh Mugele, Natasha Abadilla, Tyler Adamson, Samantha L Bernstein, Rakhee K Bhayani, Annina Elisabeth Büchi, Darcy Burbage, Christopher L Carroll, Samantha P Davis, Natasha Dhawan, Alice Eaton, Kim English, Jennifer T Grier, Mary K Gurney, Emily S Hahn, Heather Haq, Brendan Huang, Shikha Jain, Jin Jun, Wesley T Kerr, Timothy Keyes, Amelia R Kirby, Marion Leary, Mollie Marr, Ajay Major, Jason V Meisel, Erika A Petersen, Barak Raguan, Allison Rhodes, Deborah D Rupert, Nadia A Sam-Agudu, Naledi Saul, Jarna R Shah, Lisa Kennedy Sheldon, Christian T Sinclair, Kerry Spencer, Natalie H Strand, Carl G Streed Jr, Avery M Trudell

**Affiliations:** 1 School of Nursing University of Virginia Charlottesville, VA United States; 2 Northeast Georgia Medical Center Gainesville, GA United States; 3 School of Medicine Stanford University Stanford, CA United States; 4 Center for Public Health and Human Rights Department of Epidemiology Johns Hopkins School of Public Health Baltimore, MD United States; 5 College of Nursing Medical University of South Carolina Charleston, SC United States; 6 School of Medicine Washington University St. Louis, MO United States; 7 Department of General Internal Medicine, Inselspital Bern University Hospital University of Bern Bern Switzerland; 8 Oncology Nursing Consultant Newark, DE United States; 9 Department of Pediatrics University of Connecticut Farmington, CT United States; 10 Department of Respiratory Care Boise State University Boise, ID United States; 11 Hematology/Oncology Section Dartmouth-Hitchcock Medical Center Lebanon, NH United States; 12 Swansea University Medical School Swansea United Kingdom; 13 Trent/Fleming School of Nursing Peterborough, ON Canada; 14 Department of Biomedical Sciences University of South Carolina School of Medicine Greenville Greenville, SC United States; 15 College of Pharmacy Glendale Campus Midwestern University Glendale, AZ United States; 16 Department of Pediatrics Memorial Sloan Kettering Cancer Center New York, NY United States; 17 Department of Pediatrics Baylor College of Medicine Houston, TX United States; 18 Department of Neurology and Neurosurgery Tulane University New Orleans, LA United States; 19 Division of Hematology and Oncology University of Illinois Chicago Chicago, IL United States; 20 College of Nursing Ohio State University Columbus, OH United States; 21 Department of Neurology University of California Los Angeles Los Angeles, CA United States; 22 School of Nursing University of Pennsylvania Philadelphia, PA United States; 23 School of Medicine Oregon Health & Science University Portland, OR United States; 24 Section of Hematology and Oncology University of Chicago Chicago, IL United States; 25 Hunter School of Nursing City University of New York New York, NY United States; 26 University of Arkansas for Medical Sciences Little Rock, AR United States; 27 Meir Medical Center Kfar Saba Israel; 28 School of Medicine Tufts University Boston, MA United States; 29 Cold Spring Harbor Laboratory Cold Spring Harbor, NY United States; 30 State of New York-Stony Brook University Stony Brook, NY United States; 31 Institute of Human Virology and Department of Pediatrics University of Maryland School of Medicine Baltimore, MD United States; 32 International Research Center of Excellence Institute of Human Virology Nigeria Abuja Nigeria; 33 Office of Career and Professional Development University of California San Francisco San Francisco, CA United States; 34 Oncology Nursing Society Pittsburgh, PA United States; 35 University of Kansas Health System Kansas City, KS United States; 36 Department of Mathematics and Physics Stevenson University Owings Mills, MD United States; 37 Department of Anesthesiology Mayo Clinic Phoenix, AZ United States; 38 Department of Medicine School of Medicine Boston University Boston, MA United States; 39 McGovern Medical School University of Texas Health Science Center at Houston Houston, TX United States

**Keywords:** social media, promotion, tenure, health professions, scholarship, medicine, research, accomplishment, crowdsource, contribution, innovation, education, dissemination

## Abstract

**Background:**

The traditional model of promotion and tenure in the health professions relies heavily on formal scholarship through teaching, research, and service. Institutions consider how much weight to give activities in each of these areas and determine a threshold for advancement. With the emergence of social media, scholars can engage wider audiences in creative ways and have a broader impact. Conventional metrics like the h-index do not account for social media impact. Social media engagement is poorly represented in most curricula vitae (CV) and therefore is undervalued in promotion and tenure reviews.

**Objective:**

The objective was to develop crowdsourced guidelines for documenting social media scholarship. These guidelines aimed to provide a structure for documenting a scholar’s general impact on social media, as well as methods of documenting individual social media contributions exemplifying innovation, education, mentorship, advocacy, and dissemination.

**Methods:**

To create unifying guidelines, we created a crowdsourced process that capitalized on the strengths of social media and generated a case example of successful use of the medium for academic collaboration. The primary author created a draft of the guidelines and then sought input from users on Twitter via a publicly accessible Google Document. There was no limitation on who could provide input and the work was done in a democratic, collaborative fashion. Contributors edited the draft over a period of 1 week (September 12-18, 2020). The primary and secondary authors then revised the draft to make it more concise. The guidelines and manuscript were then distributed to the contributors for edits and adopted by the group. All contributors were given the opportunity to serve as coauthors on the publication and were told upfront that authorship would depend on whether they were able to document the ways in which they met the 4 International Committee of Medical Journal Editors authorship criteria.

**Results:**

We developed 2 sets of guidelines: Guidelines for Listing All Social Media Scholarship Under Public Scholarship (in Research/Scholarship Section of CV) and Guidelines for Listing Social Media Scholarship Under Research, Teaching, and Service Sections of CV. Institutions can choose which set fits their existing CV format.

**Conclusions:**

With more uniformity, scholars can better represent the full scope and impact of their work. These guidelines are not intended to dictate how individual institutions should weigh social media contributions within promotion and tenure cases. Instead, by providing an initial set of guidelines, we hope to provide scholars and their institutions with a common format and language to document social media scholarship.

## Introduction

### Background

Traditionally, promotion and tenure committees have focused on evaluating a candidate scholar’s curriculum vitae (CV), educational dossier, or portfolio to determine their academic productivity and impact. When a scholar is thought to have reached a certain threshold of productivity and impact, they are rewarded with promotion from Assistant to Associate Professor, or from Associate to full Professor. Individual institutions have always had leeway in determining thresholds for advancement, and there is wide interinstitutional variability in this determination [1]. Informally, candidates are considered ready for advancement to Associate Professor when they develop a national reputation and advancement to full Professor when they develop an international reputation. Within health professions schools (schools of medicine, nursing, public health, etc), scholarly accomplishment for the sake of promotion and tenure has typically been divided into 3 categories: teaching (eg, traditional classroom teaching, teaching lectures, invited lectures); research (eg, bench research, clinical trials, surveys, case series); and service (eg, committees, professional organization leadership, community advocacy, journal reviews). Traditionally, an individual scholar has documented productivity in research, teaching, and service by listing lectures given in certain venues, abstracts accepted at conferences, committees served on, or papers published in scientific journals. Activities within each category might have different levels of impact (ie, different weightings for the promotion and tenure committee). For example, a teaching lecture is usually considered less impactful than an invited lecture, and a grand rounds lecture at a local hospital is generally considered to be less impactful than a lecture at a national convention.

A new model of evaluating and documenting academic accomplishments was last introduced into promotion and tenure criteria at health professions schools in the United States in 1990, when Boyer [2] argued that scholarship should be reconceptualized from encompassing task-based silos to categories of discovery, integration, application, and teaching. Some schools integrated the Boyer model into their promotion and tenure review criteria, whereas others retained the traditional research, teaching, and service model. However, Boyer’s categories remained closely aligned with the task-based silos the model sought to reject, rebranding the task-based silos rather than transcending them. In this paper we refer to the categories of research, teaching, and service with the understanding that some institutions may use Boyer’s terminology instead.

Social media platforms (eg, Twitter, Facebook, Reddit, Instagram, and TikTok) now provide scholars with a significant platform for engaging in and disseminating scholarship. For example, Twitter, a social media platform with robust health researcher engagement [3,4], has over 186 million active daily users [5]. Instagram has over 1 billion active daily users [6], and TikTok has 800 million active users [7]. Each of these social media platforms has greater academic and nonacademic reach than all of the medical and nursing journals in the world combined, but none of the health professions has a widely accepted model for scholars to document their social media engagement on CVs or in promotion dossiers.

While academic engagement on social media is not free from entanglement—including lack of traditional peer review, potential for disinformation, and intermixing of nonacademic content—there remains a tremendous potential for teaching, dissemination of research, and service. Conventional metrics of academic productivity, including bibliometric indices, such as the h-index, which are now used by some institutions in promotions decisions [8-10], do not account for these new modes of scholarly engagement and dissemination. Over the past three decades, as academic engagement has become increasingly virtual, institutions have struggled to determine how to recognize and measure academic impact. How does hosting a podcast compare to teaching a semester-long course? How does publishing a page (eg, a blog post) on a highly visited website compare to publishing in an academic journal? How does advocacy on a social media site compare to advocacy within a hospital committee? Do newer metrics, such as altmetrics, that incorporate social media engagement for academic content predict future research productivity [11,12]?

### Existing Guidelines for Including Social Media in the Promotion and Tenure Process

Some institutions such as the Mayo Clinic in the United States have led the effort to recognize the scholarly impact of social media engagement and are already considering these contributions in the promotion and tenure process [13]. Similarly, the US Council of Emergency Medicine Residency Directors proposed a formal set of guidelines for including digital scholarship in this process [14]. O’Glasser et al [15] called for the recognition of social media engagement earlier in health professional careers, and provided guidance for scholars-in-training on how best to include creative and social media work in standardized applications such as the Electronic Residency Application Service for medical residencies and fellowships. Cabrera et al [12,16] and Gruzd et al [17] outlined potential categories for documentation of social media activities in the CV. Sherbino et al [18] described a consensus process for determining criteria for social media–based scholarship to inform which activities can and may be listed on a CV. Their consensus was that scholarship should be based on 4 criteria. First, the topic must be original, stemming from the authors themselves. Second, the topic should advance the field of health education through innovative theories, research, or best practices. Third, scholarship should be able to be saved, archived, and easily disseminated to the masses. Fourth, scholarship should provide an avenue for the public to comment and provide feedback in a way that elicits further discussion. Additionally, using a modified Delphi methodology to survey experts among the scientific community, Lin et al [19] identified, via an at least 90% consensus, 13 quality indicators classified into 3 domains, credibility (n=8), content (n=4), and design (n=1), that provide the groundwork to judge future social media scholarship. With a continued rise in social media use, future research should develop a method for stakeholders to structure and stratify criteria for their individual purpose. These guidelines were echoed by Shapiro et al [20], who also included recommendations related to formatting, such as including hyperlinks when relevant. Finally, Cabrera et al [16] suggested the creation of a social media portfolio similar to an education portfolio to enhance the material included in the CV.

Thus, various and sometimes divergent guidelines exist for including social media in the promotion and tenure process, but there is currently no unifying structure.

### Objective

Guidelines that are followed across institutions and are able to capture social media engagement in a meaningful way would simplify and unify communication of academic social media engagement. While there is a great deal of interinstitutional variability in how individual activities are valued, there is a pressing need for the widespread adoption of a method for documenting social media engagement in the academic CVs and dossiers of scholars in the health professions. We propose and describe a method for scholars in the health professions to cite social media activities and impact.

Before we provide these guidelines, however, we wish to issue one important caveat: scholars should neither be expected nor required to be active on social media in order to earn promotion or tenure. There are numerous reasons why someone may choose not to have a social media presence, and no scholar should be expected to disclose their reasons for making that decision. The guidelines provided in this paper should be adopted by institutions as an option for scholars to incorporate into their CV, not as a required section that must be included.

## Methods

### Framework for Open Collaboration

To create a set of unifying guidelines, we created a crowdsourced process that capitalized on the strengths of social media and generated a case example of successful use of such a medium for productive academic collaboration. In constructing a model for describing academic social media impact on a CV and in a dossier, we considered the following topics: (1) how to represent overall social media impact, (2) how to represent specific social media contributions, (3) where on the CV or dossier to list social media contributions, (4) the appropriate format for listing these contributions, and (5) how to demonstrate the reach or impact of a specific contribution.

The first author (KDA)—a tenured professor who has substantial experience writing, revising, and applying promotion and tenure criteria over the course of her career—created an initial draft of the guidelines after conducting a brief search of the literature to identify previous work describing how to cite social media impact on a CV or in the context of a promotion and tenure committee review. She then sought input from users on Twitter via a publicly accessible Google Document. There was no screening process for who could or could not join in the collaboration. Anyone who wanted to collaborate was welcome, regardless of discipline, specialty, title, country of residence, or degree completion. All contributors were given the opportunity to serve as coauthors on the publication and were told upfront that authorship would depend on whether they were able to document the ways in which they met the 4 International Committee of Medical Journal Editors (ICMJE) authorship criteria [21].

### Crowdsourced Collaboration and Consensus

Contributors edited the draft over a period of 1 week (September 12-18, 2020). The primary and secondary authors then revised the draft to make it more concise. The guidelines and manuscript were then distributed to the list of contributors for edits and adopted by the group. When including specific examples or in discussing formatting, we used Twitter as the representative social media platform because of its heavy academic health presence, and also because it was the medium used to create these recommendations. These Twitter examples should be generally applicable to other social media platforms. Content not used in the primary article was extracted and used to form the basis of a second article (unpublished data) on the benefits of social media engagement for health professionals in academia. The second article went through a similar process for editing and shared authorship.

## Results

### Proposed Model for Tracking Social Media Impact

#### Impact of Social Media

One of the primary difficulties in describing the scholarly impact of social media is that social media users and accounts do not exist in a purely academic or scholarly space, but rather encompass a spectrum between the academy, the personal, and the wider society. A social media account that highlights the latest medical studies and attempts to provide educational content, for example, may also comment on current events or post personal pictures of family or pets. Simply describing an individual’s overall social media impact may place too much impact on popularity and broad appeal rather than true scholarly impact. On the other hand, focusing on individual and specific posts ignores the impact of being able to disseminate scholarly content to a wider audience through a broad social media following [22]. That being said, public scholars benefit from building a relationship with the general public through their posts, including those that are unrelated to scholarly work. Isaacson and Looman [22] noted that social media offers a unique opportunity to provide a bridge between and across multiple networks of both laypeople and professionals, which ultimately builds social capital. It is important that promotion and tenure reviewers do not devalue a scholar’s seemingly casual interactions on social media. Establishing an authentic social media presence (“branding”) serves to further one’s reach and the subsequent engagement and dissemination of ideas.

Therefore, we propose the following guidelines for documenting scholarly impact through social media in the CV and dossier for the sake of promotion and tenure, and which attempt to incorporate the importance of overall impact and popularity, as well as the impact of individual social media posts.

#### Documenting Overall Social Media Impact on the CV

If an institution has a CV section for public scholarship (or similar), a section for social media scholarship should be inserted here. If no institutional section requirement exists, then social media scholarship may be included as a separate high-level section within the research or scholarship section. For each social media platform on which an individual is active, they should create a separate entry that includes the following: username, dates active, number of followers, and platform-specific metrics regarding overall reach (eg, total impressions on Twitter, engagement rate for YouTube channel). Scholars may choose to list whether their social media account is “verified” but, given the nontransparency of the verification process, institutions should not give significant weight to verification status.

#### Documenting Specific Social Media Contributions on the CV

High-quality and high-impact contributions that are scholarly in nature or that promote the individual scholar’s academic mission should be cited in a CV similar to citing individual publications, abstracts, lectures, or courses taught. The location for citing social media contributions will depend on the requirements of the individual institution. Some institutions require scholarly output to be divided into the traditional research, teaching, and service categories. Other institutions might divide categories into the Boyer categories or have other categories such as advocacy, dissemination, or mentorship. In this article we provide 2 different sets of guidelines that can be inserted into an institution’s dossier and CV format depending on institutional preferences.

No set threshold exists for when a scholar should include an individual social media contribution on their CV and when to exclude it. In other words, when is a social media post impactful enough to be included on one’s CV? Since scholars move between institutions over time, institutions should not attempt to define what an appropriate threshold is for listing an individual social media contribution on a scholar’s CV. Instead, the onus should be on scholars to provide a brief explanation of why they chose to include a particular social media contribution on their CV.

#### Documenting Social Media Impact in an Academic Dossier

The purpose of the academic dossier is to provide a promotion and tenure review committee with representative examples of an individual’s scholarly output and to discuss the impact of this output and how it supports their academic mission. For example, a dossier might include representative slides from a scholar’s national presentation with a discussion of why it was impactful, how many times it has been given in various venues, and how many times it has been cited by other presentations. Or a dossier might include a written curriculum for a course that a scholar taught, including a discussion about course logistics, why the approach to the material was innovative or impactful, and how many learners the course impacted. Or, as a last example, a dossier might include an example of an individual scholar’s important publications, including the number of downloads or views of that publication, awards it won, or number of times it was cited in other publications.

Similarly, scholars in the health professions should include highly impactful and representative social media contributions in their dossier. These could include contributions from any of the categories described above but should be particularly meaningful or impactful. When including social media contributions in a dossier, the scholar should include links to each contribution, screenshots, advanced metrics indicating impact (number of likes, replies, retweets, and views), and an explanation of why the scholar chose to highlight each of these contributions. In the narrative section of the dossier, the scholar should consider including a discussion of the impact of the selected social media contributions, including impact on the scholar’s future scholarly output, how the post impacted other scholars or learners, and how and when the post was cited by other posts or in other media.

To standardize the way that scholars document and present social media contributions in their CVs and dossiers, we developed 2 sets of guidelines: Guidelines for Listing All Social Media Scholarship Under Public Scholarship (in Research/Scholarship Section of CV) and Guidelines for Listing Social Media Scholarship Under Research, Teaching, and Service Sections of CV. The content of both sets of guidelines is identical, and institutions can choose which set fits their existing CV format.

### Guidelines for Listing All Social Media Scholarship Under Public Scholarship (in Research/Scholarship Section of CV)

Textbox 1 shows the first set of guidelines.

**Guidelines for Listing All Social Media Scholarship Under Public Scholarship (in Research/Scholarship Section of the Curriculum Vitae).**

**Public Scholarship -** *Social Media Scholarship*

Overall Reach (Time Period: x/x/xx to y/y/xx)

**[Platform]:** [username]

Number of Followers/Subscribers/Connections:

Number of [Tweets, Posts, Videos, etc]:

Total Impressions and/or Other Platform-Specific Metrics:

**Select Social Media Contributions**

**Innovation** (contributions that propose new ideas)

     Link to contributions:

     Number of impressions:

     Explanation of why the scholar chose to highlight this:

**Dissemination** (contributions that share resources and/or findings)

     Link to contributions:

     Number of impressions:

     Explanation of why the scholar chose to highlight this:

**Education** (contributions that teach people something)

     Link to contribution:

     Number of impressions:

     Explanation of why the scholar chose to highlight this:

**Advocacy** (contributions about changing laws, policies, practices, and/or systems)

     Link to contribution:

     Number of impressions:

     Explanation of why the scholar chose to highlight this:

**Mentorship** (contributions about mentees achievements)

     Link to contribution:

     Number of impressions:

     Explanation of why the scholar chose to highlight this contribution:

     Presentations/Chats/Blogs/Podcasts Delivered Via Social Media

**[Platform] Chats**

     Name of [Platform] Chat:

     Role: [host, cohost, etc]

     If longitudinal, list time period and cite metrics. If discrete, list dates and topics:

     Explanation of why the scholar chose to highlight this contribution:

**[Platform] Live Video**

     Host or cohost(s) of live feed:

     Individual sessions [Date(s), Topic(s), Engagement/Reach]:

     Permanent Link:

     Explanation of why the scholar chose to highlight this contribution:

**[Platform] Recorded Video**

     Host or cohost(s) of recorded video:

     Title of Video:

     Date(s), Topic(s), Engagement/Reach:

     Permanent Link:

     Explanation of why the scholar chose to highlight this contribution:

**Blog Posts**

     Author:

     Title of Blog:

     Organization/Entity Publishing the Blog:

     Date Published:

     Link to Blog:

     Explanation of why the scholar chose to highlight this contribution:

**Podcasts**

     Role: [Host or Guest]

     Name of Podcast:

     Date Released:

     Link to Podcast Episode:

     Explanation of why the scholar chose to highlight this contribution:

     Infographics/Other Visuals

**Infographic**

     Title of Infographic:

     Date Posted/Published:

     Link to Infographic:

     Explanation of why the scholar chose to highlight this contribution:

**Other Visuals**

     Author/Creator:

     Title of Visual:

     Date Posted/Published:

     Link to Visual:

     Explanation of why the scholar chose to highlight this contribution:

### Guidelines for Listing Social Media Scholarship Under Research, Teaching, and Service Sections of CV

Textbox 2 shows the second set of guidelines.

**Guidelines for Listing Social Media Scholarship Under Research, Teaching, and Service Sections of the Curriculum Vitae.**

**RESEARCH -** *Social Media Scholarship*

Overall Reach (Time Period: x/x/xx to y/y/xx)

**[Platform]:** [username]

Number of Followers/Subscribers/Connections:

Number of [Tweets, Posts, Videos, etc]:

Total Impressions and/or Other Platform-Specific Metrics:

**Select Social Media Contributions**

**Innovation** (contributions that propose new ideas)

     Link to contributions:

     Number of impressions:

     Explanation of why the scholar chose to highlight this:

**Dissemination** (contributions that share resources and/or findings)

     Link to contributions:

     Number of impressions:

     Explanation of why the scholar chose to highlight this

     Scholarly Presentations/Chats/Blogs/Podcasts Delivered Via Social Media

**[Platform] Chats**

     Name of [Platform] Chat:

     Role: [host, cohost, etc]

     If longitudinal, list time period and cite metrics:

     If discrete, list dates and topics:

     Explanation of why the scholar chose to highlight this contribution:

**[Platform] Live Video**

     Host or cohost(s) of live feed:

     Individual sessions [Date(s), Topic(s), Engagement/Reach]:

     Permanent Link:

     Explanation of why the scholar chose to highlight this contribution:

**[Platform] Recorded Video**

     Host or cohost(s) of recorded video:

     Title of Video:

     Date(s), Topic(s), Engagement/Reach:

     Permanent Link:

     Explanation of why the scholar chose to highlight this contribution:

**Blog Posts**

     Author:

     Title of Blog:

     Organization/Entity Publishing the Blog:

     Date Published:

     Link to Blog:

     Explanation of why the scholar chose to highlight this contribution:

**Podcasts**

     Role: [Host or Guest]

     Name of Podcast:

     Date Released:

     Link to Podcast Episode:

     Explanation of why the scholar chose to highlight this contribution:

     Scholarly Infographics/Other Visuals

**Infographic**

     Title of Infographic:

     Date Posted/Published:

     Link to Infographic:

     Explanation of why the scholar chose to highlight this contribution:

**Other Visuals**

     Author/Creator:

     Title of Visual:

     Date Posted/Published:

     Link to Visual:

     Explanation of why the scholar chose to highlight this contribution:

**TEACHING**

**Select Social Media Contributions**

**Education** (contributions that teach people something)

     Link to contribution:

     Number of impressions:

     Explanation of why the scholar chose to highlight this:

     Educational Presentations/Chats/Blogs/Podcasts Delivered Via Social Media

**[Platform] Chats**

     Name of [Platform] Chat:

     Role: [host, cohost, etc]

     If longitudinal, list time period and cite metrics. If discrete, list dates and topics:

     Explanation of why the scholar chose to highlight this contribution:

**[Platform] Live Video**

     Host or cohost(s) of live feed:

     Individual sessions [Date(s), Topic(s), Engagement/Reach]:

     Permanent Link:

     Explanation of why the scholar chose to highlight this contribution:

**[Platform] Recorded Video**

     Host or cohost(s) of recorded video:

     Title of Video:

     Date(s), Topic(s), Engagement/Reach:

     Permanent Link:

     Explanation of why the scholar chose to highlight this contribution:

**Blog Posts**

     Author:

     Title of Blog:

     Organization/Entity Publishing the Blog:

     Date Published:

     Link to Blog:

     Explanation of why the scholar chose to highlight this contribution:

**Podcasts**

     Role: [Host or Guest]

     Name of Podcast:

     Date Released:

     Link to Podcast Episode:

     Explanation of why the scholar chose to highlight this contribution:

     Educational Infographics/Other Visuals

**Infographic**

     Title of Infographic:

     Date Posted/Published:

     Link to Infographic:

     Explanation of why the scholar chose to highlight this contribution:

**Other Visuals**

     Author/Creator:

     Title of Visual:

     Date Posted/Published:

     Link to Visual:

     Explanation of why the scholar chose to highlight this contribution:

**SERVICE**

**Select Social Media Contributions**

**Advocacy** (contributions about changing laws, policies, practices, and/or systems)

     Link to contribution:

     Number of impressions:

     Explanation of why the scholar chose to highlight this:

**Mentorship** (contributions about mentees achievements)

     Link to contribution:

     Number of impressions:

     Explanation of why the scholar chose to highlight this contribution.

## Discussion

### Limitations of the Guidelines

In developing these guidelines, we attempted to account for the breadth of social media platforms, as well the variability in institutional guidelines and expectations for promotion and tenure. Given the growing list of social media platforms and the wide variety of ways in which users can engage with them, developing a set of immutable guidelines was neither possible nor practical. Social media platforms will continue to evolve, and new platforms will continue to be introduced that provide novel ways for health scholars to interact with each other and the public, but will continue to raise new challenges about the scope and nature of academic impact on these platforms.

Current metrics for measuring the impact of social media use, whether an individual’s overall social media impact or the impact of a specific contribution or facet of a scholar’s social media presence, rely on blunt and nonspecific markers of impact, including numbers of followers, numbers of likes and shares, and other social media users’ amount of engagement. Our hope is that as research continues to show the general impact of social media on health scholarship, more refined metrics for measuring individual impacts will become apparent.

We recognize that some institutions remain unsure of the relative impact of social media scholarship in the academic realm. For the purpose of developing these guidelines, we specifically chose not to discuss the merits of social media in this paper, but rather to assume that scholarly impact within social media is worthwhile and worthy of inclusion in the promotion and tenure process.

### Conclusion

This paper presents crowdsourced guidelines on how to cite scholarly productivity on social media, while recognizing the limitations inherent in measuring social media impact across a variety of academic institutions. These guidelines describe a process and structure for documenting and describing a scholar’s general impact on a social media platform, as well as methods of documenting individual social media contributions such as teaching posts, mentorship, and ongoing advocacy.

Since promotion and tenure is decided by individual academic institutions based on the criteria developed and adopted by their faculty, these guidelines are not intended to dictate how individual institutions should weigh social media contributions within promotion and tenure cases. Instead, by providing an initial set of guidelines, we hope to provide scholars and their institutions with a common format and language to describe what is becoming more and more ubiquitous among academics.

We expect that as social media adoption and use continues to grow among academics, and as new social media platforms arise and new methods of applying social media to health education and scholarly distribution become apparent, research will continue to demonstrate the impact of social media on education, potentially affecting the scope of distribution, health care advocacy and equity in traditionally inequitable fields (such as gender and race), and scholarly innovation.

## Abbreviations

CV: curriculum vitae
ICMJE: International Committee of Medical Journal Editors
