# Correction: Documenting Social Media Engagement as Scholarship: A New Model for Assessing Academic Accomplishment for the Health Professions

**DOI:** 10.2196/26225

**Published:** 2020-12-14

**Authors:** Kimberly D Acquaviva, Josh Mugele, Natasha Abadilla, Tyler Adamson, Samantha L Bernstein, Rakhee K Bhayani, Annina Elisabeth Büchi, Darcy Burbage, Christopher L Carroll, Samantha P Davis, Natasha Dhawan, Alice Eaton, Kim English, Jennifer T Grier, Mary K Gurney, Emily S Hahn, Heather Haq, Brendan Huang, Shikha Jain, Jin Jun, Wesley T Kerr, Timothy Keyes, Amelia R Kirby, Marion Leary, Mollie Marr, Ajay Major, Jason V Meisel, Erika A Petersen, Barak Raguan, Allison Rhodes, Deborah D Rupert, Nadia A Sam-Agudu, Naledi Saul, Jarna R Shah, Lisa Kennedy Sheldon, Christian T Sinclair, Kerry Spencer, Natalie H Strand, Carl G Streed Jr, Avery M Trudell

**Affiliations:** 1 School of Nursing University of Virginia Charlottesville, VA United States; 2 Northeast Georgia Medical Center Gainesville, GA United States; 3 School of Medicine Stanford University Stanford, CA United States; 4 Center for Public Health and Human Rights Department of Epidemiology Johns Hopkins School of Public Health Baltimore, MD United States; 5 College of Nursing Medical University of South Carolina Charleston, SC United States; 6 School of Medicine Washington University St. Louis, MO United States; 7 Department of General Internal Medicine, Inselspital Bern University Hospital University of Bern Bern Switzerland; 8 Oncology Nursing Consultant Newark, DE United States; 9 Department of Pediatrics University of Connecticut Farmington, CT United States; 10 Department of Respiratory Care Boise State University Boise, ID United States; 11 Hematology/Oncology Section Dartmouth-Hitchcock Medical Center Lebanon, NH United States; 12 Swansea University Medical School Swansea United Kingdom; 13 Trent/Fleming School of Nursing Peterborough, ON Canada; 14 Department of Biomedical Sciences University of South Carolina School of Medicine Greenville Greenville, SC United States; 15 College of Pharmacy Glendale Campus Midwestern University Glendale, AZ United States; 16 Department of Pediatrics Memorial Sloan Kettering Cancer Center New York, NY United States; 17 Department of Pediatrics Baylor College of Medicine Houston, TX United States; 18 Department of Neurology and Neurosurgery Tulane University New Orleans, LA United States; 19 Division of Hematology and Oncology University of Illinois Chicago Chicago, IL United States; 20 College of Nursing Ohio State University Columbus, OH United States; 21 Department of Neurology University of California Los Angeles Los Angeles, CA United States; 22 School of Nursing University of Pennsylvania Philadelphia, PA United States; 23 School of Medicine Oregon Health & Science University Portland, OR United States; 24 Section of Hematology and Oncology University of Chicago Chicago, IL United States; 25 Hunter School of Nursing City University of New York New York, NY United States; 26 University of Arkansas for Medical Sciences Little Rock, AR United States; 27 Meir Medical Center Kfar Saba Israel; 28 School of Medicine Tufts University Boston, MA United States; 29 Cold Spring Harbor Laboratory Cold Spring Harbor, NY United States; 30 State of New York-Stony Brook University Stony Brook, NY United States; 31 Institute of Human Virology and Department of Pediatrics University of Maryland School of Medicine Baltimore, MD United States; 32 International Research Center of Excellence Institute of Human Virology Nigeria Abuja Nigeria; 33 Office of Career and Professional Development University of California San Francisco San Francisco, CA United States; 34 Oncology Nursing Society Pittsburgh, PA United States; 35 University of Kansas Health System Kansas City, KS United States; 36 Department of Mathematics and Physics Stevenson University Owings Mills, MD United States; 37 Department of Anesthesiology Mayo Clinic Phoenix, AZ United States; 38 Department of Medicine School of Medicine Boston University Boston, MA United States; 39 McGovern Medical School University of Texas Health Science Center at Houston Houston, TX United States

In “Documenting Social Media Engagement as Scholarship: A New Model for Assessing Academic Accomplishment for the Health Professions” (J Med Internet Res 2020;22(12):e25070), one of the authors was not mentioned in the original paper.

The following author has been added:

Alice Eaton, Bsc

Swansea University Medical School, Swansea, United Kingdom

One of the contributors was missed in the Acknowledgement section. Previously, the Acknowledgement section read as follows:

All authors met the ICMJE authorship criteria, including reviewing and approving this manuscript for publication. Each of the 40 coauthors completed an Authorship Attestation Form confirming that they met all 4 of the ICMJE authorship criteria. In addition, each coauthor described on a shared Google Sheet how they made “substantial contributions to conception and design, or acquisition of data, or analysis and interpretation of data.” The authors wish to thank Jocelyn C Anderson, PhD, RN, S Alexander Kemery, PhD, RN, and Chrystal L Lewis, PhD, RN for their comments on the draft of this manuscript.

The corrected Acknowledgement section now reads as follows:

All authors met the ICMJE authorship criteria, including reviewing and approving this manuscript for publication. Each of the 40 coauthors completed an Authorship Attestation Form confirming that they met all 4 of the ICMJE authorship criteria. In addition, each coauthor described on a shared Google Sheet how they made “substantial contributions to conception and design, or acquisition of data, or analysis and interpretation of data.” The authors wish to thank Jocelyn C Anderson, PhD, RN, S Alexander Kemery, PhD, RN, Rebecca Koszalinski PhD, RN, CRRN, CMSRN, and Chrystal L Lewis, PhD, RN for their comments on the draft of this manuscript.

The affiliation for authors Brendan Huang and Ajay Major was incorrect. They were displayed as:

Brendan Huang

Department of Clinical Neurosciences, Tulane University, New Orleans, LA, United States

Ajay Major

University of Chicago, Section of Hematology and Oncology, Chicago, IL, United States

They have been updated to:

Brendan Huang

Department of Neurology and Neurosurgery, Tulane University, New Orleans, LA, United States

Ajay Major

Section of Hematology and Oncology, University of Chicago, Chicago, IL, United States

The correction will appear in the online version of the paper on the JMIR Publications website on December 9, 2020, together with the publication of this correction notice. Because this was made after submission to PubMed, PubMed Central, and other full-text repositories, the corrected article has also been resubmitted to those repositories.

